# How Adult Child Caregivers Distribute Care Tasks for Their Parents Within a Care Network

**DOI:** 10.1093/geroni/igaf122.2654

**Published:** 2025-12-31

**Authors:** Eunju Lee, Haeun Yoo, Jiweon Jun, Kyungmin Kim

**Affiliations:** Seoul National University, Seoul, Republic of Korea; Seoul National University, Seoul, Republic of Korea; Seoul National University, Seoul, Republic of Korea; Seoul National University, Seoul, Republic of Korea

## Abstract

Primary caregivers of older adults often share caregiving responsibilities with family or paid caregivers. However, less is known about how care network members engage in different roles, how tasks are distributed (e.g., jointly performed by the primary caregiver and others or solely handled by others), and which way of performing tasks is more beneficial to caregivers. This study examined how adult child caregivers perform care tasks with care network members and its association with positive and negative caregiving experiences. Adult children who are primary caregivers of their older parents/in-law (N = 767; aged 28–69) reported their parents’ care needs in 16 activities and who performed each task. Among activities that need assistance, 65% were jointly managed by the primary caregiver and others, 18% were handled solely by the primary caregiver, and 17% solely by others. Besides the primary caregiver, the older parent’s daughters performed various daily activities, while sons offered intermittent, mobility-focused assistance (e.g., medical visits); the parent’s daughters-in-law were less likely to help with activities requiring physical contact (e.g., bathing), and paid caregivers mainly helped with parents’ activities of daily living. Regression analyses showed that caregivers reported less caregiving stress and more personal time—when more tasks were handled solely by other family members. However, caregivers experienced more interference while caregiving—when they jointly managed more tasks with family members. Having more tasks solely managed by paid caregivers was associated with lower positive gain. The findings highlight the importance of considering ways of distributing care tasks for caregivers’ well-being.

